# Oligomerisation Drives Internal Cavity Formation and Conformational Heterogeneity in Mixed Triton X‐100/Tyloxapol Micelles

**DOI:** 10.1002/smsc.70376

**Published:** 2026-08-22

**Authors:** Hrachya Ishkhanyan, David J. Barlow, M. Jayne Lawrence, Christian D. Lorenz

**Affiliations:** ^1^ Computational Molecular Engineering Lab Institute for Informatics and Automation Problems of the National Academy of Sciences of the Republic of Armenia Yerevan Republic of Armenia; ^2^ Biological & Soft Matter Research Group Department of Physics Faculty of Natural Mathematical & Engineering Sciences King’s College London London UK; ^3^ Division of Pharmacy and Optometry School of Health Sciences Faculty of Biology Medicine and Health University of Manchester Stopford Building Manchester UK; ^4^ The University of Manchester at Harwell Diamond Light Source Harwell Campus Didcot Oxfordshire UK; ^5^ Department of Engineering Faculty of Natural Mathematical & Engineering Sciences King’s College London London UK

**Keywords:** chemistry, critical micelle concentration, micelle, molecular dynamics, neutron scattering, oligomer, pulmonary surfactant, stacking, trimer

## Abstract

Oligomeric surfactants exhibit physicochemical properties distinct from their monomeric counterparts, including lower critical micelle concentrations, yet how oligomerisation shapes the internal architecture of the micelles they form remains poorly understood. Here, we combine all‐atom molecular dynamics simulations with small‐angle neutron scattering (SANS) to study mixed micelles of Triton X‐100 and its oligomeric analogue Tyloxapol (trimer and heptamer) across eight compositions spanning pure Triton X‐100 to pure Tyloxapol. Tyloxapol content and oligomer length control the internal organization of the micelles: Tyloxapol‐rich micelles adopt compact, oblate structures containing internal, water‐filled cavities formed by headgroup penetration into the hydrophobic core, accompanied by reduced *π*–*π* stacking between aromatic tail groups. For pure Tyloxapol micelles, SANS corroborates the simulated size, aggregation number, oblate shape, and core hydration. Neighbor analysis shows no preferential association between Triton X‐100 and Tyloxapol, consistent with random mixing across all compositions studied, while unsupervised clustering (UMAP/HDBSCAN) of Tyloxapol conformations shows that the local micelle environment selects for distinct conformational states. Together, these findings identify surfactant oligomerisation as a route to controlling internal micelle architecture, rather than only overall micelle size and shape, with implications for the design of nanocarriers and functional soft materials with tunable internal structure.

## Graphical abstract

Surfactant oligomerisation reshapes mixed micelles beyond changes in size and shape. Molecular simulations and neutron scattering reveal that Tyloxapol promotes water‐filled internal cavities, heterogeneous conformations, and compact oblate architectures, demonstrating how oligomer design provides a route to engineer the internal structure of soft nanomaterials.

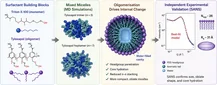

## 1 Introduction

Surfactants are amphiphilic molecules that can self‐assemble in aqueous environments into a variety of structures—including spherical micelles, rod‐like aggregates, and vesicles—depending on their molecular architecture and concentration. Due to their ability to self‐assemble, lower surface tension, and wet surfaces, surfactants are widely used across industries, including cosmetics, agriculture, and pharmaceuticals, as well as in cleaning products and detergents [1, 2, 3, 4]. Surfactants are commonly classified as anionic (negatively charged), cationic (positively charged), zwitterionic (bearing both positive and negative charges), or nonionic (uncharged). Nonionic surfactants are generally favored for formulation purposes due to their lower toxicity and stability under various conditions. Among nonionic surfactants, Triton X‐100 and its oligomeric counterpart, Tyloxapol, have been the focus of extensive study.

Oligomeric surfactants—typically synthesized by covalently linking monomeric surfactants via a spacer—have attracted increasing interest, as they often exhibit physicochemical properties distinct from their monomeric analogues [5, 6, 7]. These properties can be fine‐tuned by modifying the length and chemical nature of the hydrophobic tails, the structure of the linker (e.g., linear or branched), and the number of surfactant units joined together, enabling a broad range of structural variants.

Several studies have reported that reducing the length of the spacer between surfactant units decreases both the critical micelle concentration (CMC) and surface tension, while also improving foaming behavior. This behavior has been observed for dimeric cationic surfactants joined via their head group (often referred to as gemini surfactants) derived from dodecyl‐ or benzyldodecyl‐trimethylammonium chloride [5]. Indeed, dimeric and trimeric surfactants generally exhibit lower CMCs and surface tensions than their monomeric counterparts [6, 7, 8, 9, 10]. For example, Bunton et al. reported a reduced CMC for a dicationic analog of the cationic surfactant cetyltrimethylammonium bromide (CTABr) [9]. Zhou et al. demonstrated enhanced antimicrobial activity in ammonium surfactants as the degree of oligomerization increased, with trimeric, tetrameric, and hexameric species showing significantly greater activity against *E. coli* than their monomeric or dimeric forms [10].

In structurally related surfactant series, lower CMC values are generally associated with larger micelles and increased solubilization capacity. For instance, Dharaiya et al. compared the solubilization of poorly water‐soluble bisphenol A in micelles formed by Tyloxapol and Triton X‐100, finding that Tyloxapol formed the largest micelle and exhibited the highest solubilization capacity [11]. Similarly, Dam et al. reported that dimeric quaternary ammonium bromides possessed both lower CMCs and greater oil solubilization capacity than the corresponding monomeric surfactant, CTABr [12], an experimental observation corroborated by coarse‐grain molecular dynamics (MD) simulations. Maiti et al. used coarse‐grained MD simulations to show that increasing the degree of polymerization in a surfactant lowers the CMC [13]. A similar trend is observed in dissipative particle dynamics simulations of SDS [14]. More recently, Wang et al. investigated the effect of spacer length on the morphology of micelles formed by dimeric dimethylcetylammonium bromide surfactants, observing a transition from micelles to vesicles as the spacer length decreased [15].

In some cases, micelles and other nanostructures are formed from mixtures of surfactants rather than from a single species [16]. Depending on the surfactants involved, mixed micellar systems may outperform their single‐component counterparts across a range of applications [16, 17, 18]. The CMCs of mixed micellar systems typically lie between those of the individual surfactants [17]. Various physicochemical properties, including micelle size, shape, interaction parameters, and CMC, have been systematically explored in mixed systems [19, 20, 21, 22]. Mixed micelles have also received attention for their ability to enhance the solubility of poorly water‐soluble drugs and to improve the therapeutic effect of the drugs they contain [23]. Experimental studies have demonstrated that two‐component micelles can solubilize more doxorubicin, an antitumor drug, and enhance its therapeutic efficacy [24, 25, 26, 27]. For example, Mehta et al. [28] showed that lecithin‐Tyloxapol micelles exhibit synergistic behavior and a high drug‐encapsulation capacity. The self‐assembly of binary surfactant mixtures has also been investigated through atomistic and coarse‐grained MD simulations [29, 30, 31], including systems based on CTAB, SDS, and glycocholate. However, to date, no computational studies have explored mixed micelles composed of a surfactant and its oligomer. This leaves several fundamental questions: (1) How does cross‐linking between surfactant molecules affect micelle formation? (2) How do micellar morphologies differ among assemblies formed by monomeric surfactants, oligomeric surfactants, and their mixtures?

**FIGURE 1:**
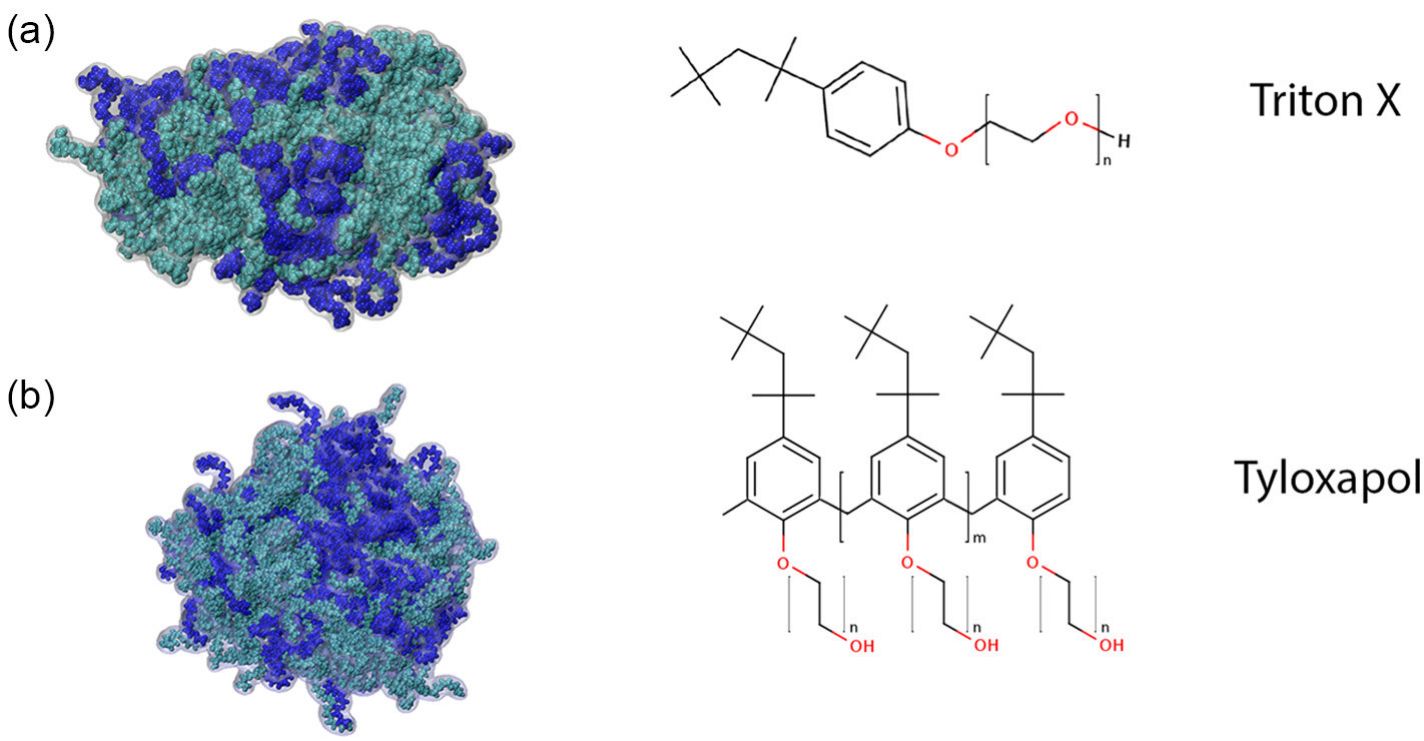
Molecular architectures of Triton X‐100, Tyloxapol, and their mixed micelles as observed in the simulations. (a) Representative snapshots of 50:50 mixed micelles composed of Triton X‐100 and Tyloxapol oligomers of two different lengths: m=3 (TY39, top) and m=7 (TY79, bottom). (b) Chemical structures of Triton X‐100 (n≈9.5, top) and Tyloxapol (m≈7, bottom), highlighting the aromatic core and the PEG‐based headgroups characteristic of these nonionic surfactants.

In this study, we investigate the structure of mixed micelles formed from Triton X‐100 and Tyloxapol (Figure 1b). Triton X‐100 consists of a hydrophilic polyethylene oxide (PEO) head group and a hydrophobic 4‐(1,1,3,3‐tetramethylbutyl)‐phenyl tail. Tyloxapol is an oligomeric analogue of Triton X‐100, composed of approximately seven monomer units covalently linked via a spacer. In this manuscript, we have studied variants of Tyloxapol that have 3 and 7 monomer units, and each have the same number, 10, of ethylene oxide groups in their hydrophilic headgroups as the Triton X‐100 molecules that have been modeled, the nearest integer to the experimentally reported average of *n* ∼ 9.5 (Figure 1a). We explore the structural properties of mixed micelles formed at varying ratios of Triton X‐100 and Tyloxapol (Figure 1a), and assess the effects of cross‐linking on micelle morphology and structural properties.

## 2 Methods

### 2.1 Molecular Dynamics Simulations

In this work, mixed Triton X‐100 and Tyloxapol micelles have been investigated. All‐atom MD simulations have been performed using the GROMACS package [32, 33, 34] and the CHARMM36 [35, 36, 37] force field. Triton X‐100 is modeled using CHARMM parameters reported by Yordanova et al. [38]. Two types of Tyloxapol molecules have been constructed, a trimer (TY39) and a Triton X‐100 (TX100) heptamer (TY79) (Figure 1b), and were parameterized by the CHARMM36 general force field [39]. The water is modeled using CHARMM36m TIP3 potential.

In total, eight systems, containing varying amounts of Triton X‐100 and Tyloxapol, have been constructed and simulated. In Table 1, the number and type of species in each system is presented along with their abbreviated description. The amounts of both surfactants were chosen such that the total number of Triton X‐100 monomer units, counting each Tyloxapol oligomer as its constituent TX100 subunits (3 for TY39, 7 for TY79) is approximately 150. This value was chosen to match the experimentally reported aggregation number of Triton X‐100 micelles, which falls in the range of 100–155, and to enable direct comparison with our previous simulations of pure Triton X‐100 micelles [40]. The total monomer count was held fixed across all eight systems so that composition and oligomer length are the only variables governing differences in the resulting micelle structure; composition‐dependent equilibrium aggregation numbers were not determined and are identified as a direction for future work (see Section 4). First, the surfactants in the system were preassembled into a spherical “micelle‐like” structure using the Packmol software [41]. The “micelles” were then centered in a simulation box with dimensions of 120 Å × 120 Å × 120 Å, after which water was added to fill the box.

**TABLE 1:** Composition of the eight simulation systems. Listed are the percentages and total number of each surfactant species (Tyloxapol—TY and Triton X‐100—TX100) in the systems that have been simulated. Note that Tyloxapol consisting of three (TY39) and seven monomers (TY79) and each has been studied. The number of water molecules are included for completeness. The total number of Triton X‐100 monomers (N) found in the Tyloxapol and Triton X‐100 surfactants in each simulated system is also reported.

System	TY, %	TX100, %	TY, N	TX100, N	Water	Monomer, *N*
TY39	100	0	50	0	51 463	150
TY39‐TX100 75	75	25	38	38	51 289	152
TY39‐TX100 50	50	50	25	75	51 237	150
TY39‐TX100 25	25	75	13	112	51 084	151
TY79	100	0	21	0	51 373	147
TY79‐TX100 75	75	25	16	38	51 158	150
TY79‐TX100 50	50	50	11	75	51 242	152
TY79‐TX100 25	25	75	5	113	51 041	148

Within each preassembled structure, Triton X‐100 and Tyloxapol molecules were placed by Packmol at random positions and orientations, subject only to a minimum‐distance overlap constraint, so that the mixed micelles are randomly, not deliberately, mixed at the outset. The radius of the starting sphere was chosen to approximate the packing density of a Triton X‐100 micelle of comparable aggregation number reported in our previous work, giving a starting structure that is close to, but not fixed at, the expected equilibrium density. The subsequent energy minimization and NVT/NPT equilibration and production stages allow both the local packing and the overall micelle/box density to relax freely under the Parrinello–Rahman barostat. The size, shape, and volume of each system as a function of simulation time are shown in Figures S1–S3; all systems show substantial relaxation away from their initial Packmol values within the first ∼100–300 ns, after which each descriptor plateaus, which we take as evidence that the systems are not trapped near their initial, unrelaxed configuration.

The steepest descent algorithm is applied to minimize the energy in each system. The systems were then brought to the desired temperature of 303.15 K in a canonical, NVT, ensemble using a Nosé–Hoover thermostat, after which the systems were equilibrated in an NPT ensemble to achieve a target pressure of 1 bar, coupled to a Nosé–Hoover thermostat and Parrinello–Rahman barostat. The time constants for the thermostat and barostat were set to 1 and 5 ps, respectively, with isotropic pressure coupling applied to the barostat. The durations for the equilibration simulations were set to 75 and 325 ps for the NVT and NPT simulations, respectively. Then each system is simulated for 600 ns in an NPT ensemble using a timestep of 2 fs. The TY39‐TX100 75 system did not fully stabilize within the standard run time and was therefore extended to 800 ns. The LINCS algorithm [42] is applied to constrain hydrogen‐containing bonds. A cutoff of 1.2 nm is used to truncate short‐range interactions. Long‐range interactions were calculated using the Particle‐Mesh Ewald algorithm.

### 2.2 Analysis of MD Simulations

**FIGURE 2:**
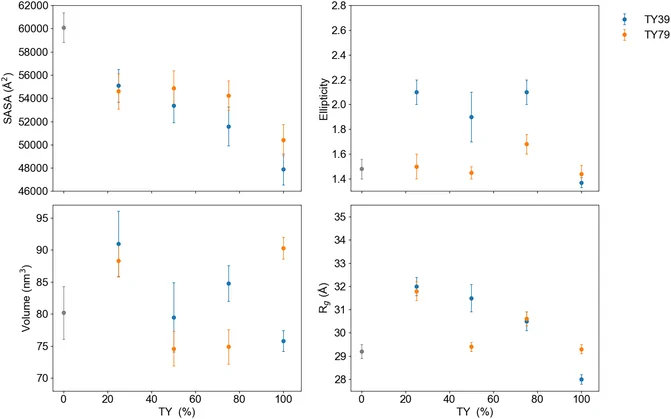
Key global structural descriptors of Tyloxapol‐containing micelles obtained from our molecular dynamics simulations as a function of Tyloxapol composition. Plotted are the average solvent‐accessible surface area (SASA), ellipticity, volume, and radius of gyration versus the percentage of Tyloxapol present in the micelle. Gray circles indicate the corresponding average values for 100% Triton X‐100 micelles from our previous work [40], providing a reference point for assessing how increasing Tyloxapol content influences micelle size, anisotropy, and overall morphology.

The analysis of the systems is conducted using tools provided by the GROMACS software package as well as in‐house Python scripts. The calculated values for each parameter were averaged over the portion of the trajectory where the system has reached its stability determined by the root‐mean‐square deviation (RMSD) of the coordinates of the atoms within the molecules constituting the micelle. Uncertainties reported throughout (Table 2, Figure 2, and elsewhere) represent the standard deviation.

**TABLE 2:** Global structural and morphological descriptors obtained from the molecular dynamics simulations of the Triton X‐100 and tyloxapol micelles. The table includes the average solvent‐accessible surface area (SASA), ellipticity, volume, and radius of gyration for each system, together with the corresponding nanoparticle shape classifications. These metrics provide a comparative view of overall micelle size, compactness, and anisotropy, enabling direct assessment of how tyloxapol composition and oligomer length influence micelle morphology and structural stability.

System	SASA (Å^2^)	Ellipticity	Volume, nm^3^	*R*_g_, Å	Shape
TY39	47887±1335	1.370±0.04	75.8±1.6	28.03±0.2	Oblate
TY39‐TX100 75	51581±1658	2.1±0.1	84.8±2.8	30.5±0.4	Triaxial
TY39‐TX100 50	53353±1462	1.902±0.2	79.5±5.4	31.50±0.6	Prolate
TY39‐TX100 25	55087±1400	2.1099±0.1	91.0±5.1	32.02±0.4	Prolate
TY79	50409±1359	1.4438±0.07	90.3±1.7	29.3±0.2	Oblate
TY79‐TX100 75	54235±1260	1.682±0.08	74.9±2.7	30.64±0.3	Prolate
TY79‐TX100 50	54884±1478	1.453±0.05	74.6±2.7	29.437±0.2	Oblate
TY79‐TX100 25	54590±1512	1.5496±0.1	88.3±2.5	31.877±0.4	Prolate
TX100	60100±1279	1.48±0.08	80.2±4.1	29.215±0.3	Prolate

#### 2.2.1 Size and Shape of Micelles

To determine the stable micelle structure, the RMSD and the radius of gyration have been calculated. Furthermore, the solvent‐accessible surface area (SASA) is calculated to assess the surface area of each aggregate.

To determine the shape of the micelle, the inertia tensor is calculated and averaged over the last 200 frames of the trajectory, saved every 100 ps, corresponding to the final 20 ns. This window was chosen because it lies within the plateau region reached by all global size/shape descriptors (Figures S1–S4), typically after the first 100–300 ns of simulation. The micelle shape can be classified as spherical, oblate, prolate, or triaxial. The criterion for a micelle to be classified as a certain shape is described in terms of the principal moments of inertia ($I_{1}$, $I_{2}$, and $I_{3}$) as follows:

1. $I_{1}≃I_{2}≃I_{3}$, micelle is spherical.
2. $I_{1}≃I_{2}>I_{3}$, micelle is oblate.
3. $I_{1}≃I_{2}<I_{3}$, micelle is prolate.
4. $I_{1}\neq I_{2}\neq I_{3}$, micelle is triaxial.

The moments of inertia can be used further to quantify the shape by computing the ellipticity parameter, ɛ, which is defined as

(1) $$ɛ=\frac{I_{\max}}{I_{\min}}$$

where $I_{\max}$ and $I_{\min}$ are the largest and smallest principal moments of inertia, meaning that the structure is a perfect sphere when ɛ=1, and increases as the structure becomes less spherical.

#### 2.2.2 Interface Definition and Density Calculation

The hydrophobic core–water interface is defined using our recently published Python package, Python Utility for Characterizing Heterogeneous Interfaces and Kinetics (PUCHIK) [43]. PUCHIK constructs the interface by forming a convex hull around user‐defined atom groups and then divides the simulation box into a grid. The distance from the center of each grid cell to the hydrophobic core–water interface is calculated, with the reference point r=0 being any point on the hull. Note that this reference point is not the center of the nanoparticle. The density calculations are then straightforward and are estimated for each individual cell of the grid.

#### 2.2.3 Neighbor Analysis

Graph theory has been applied to identify the nearest neighbor molecules around a given molecule and determine their mutual connectivity. In doing so, an adjacency matrix is constructed as follows. First, the pairwise distance matrix between centere of mass of the surfactant molecules is calculated. If the distance between two molecules (i and j) is less than the cutoff ($r_{c}=10$ Å), then the element representing the interaction of molecules i and j within the adjacency matrix is set to 1, otherwise the element is given a value of 0. This is done for all unique pairs of molecules i and j such that the elements of the adjacency matrix are defined as

(2) $$a_{ij}=\left\{ \begin{array}{c} 1\;\text{if }r_{ij}\leq r_{c}\\ 0\;\text{if }r_{ij}>r_{c} \end{array}\right.$$

where $r_{ij}$ is the distance between particles i and j. Afterward, for each molecule, types and quantities of neighbors are found, and are used to calculate the ratio $\frac{N_{a}}{N_{\mathrm{tot}}}$ and $\frac{N_{b}}{N_{\mathrm{tot}}}$, where $N_{a}$ and $N_{b}$ are the numbers of neighbors of type a and type b of the molecule, respectively, and $N_{\mathrm{tot}}$ is the total number of neighbors.

#### 2.2.4 Analyzing Conformations

To understand the internal structure of the resultant micelles, we performed analysis to distinguish the various conformations of the Tyloxapol molecules throughout the trajectory. To do this, four atoms in the hydrophobic tail of Tyloxapol were selected, and the pairwise distances between the atoms were computed. An unsupervised machine learning approach (UMAP [44]) is used to reduce the dimensionality of the data which is then clustered using the HDBSCAN [45] algorithm. Each cluster represents a separate conformation. A similar approach has been previously employed to successfully identify unique conformations in polymers [46, 47].

### 2.3 Small‐Angle Neutron‐Scattering (SANS) Experiments

SANS experiments were used to establish the structure of tyloxapol micelles dispersed in $D_{2}O$ at concentrations of 1, 5, 10, and 15 weight%. The SANS experiments were performed using the instruments SANS2D at the ISIS‐pulsed neutron source (Rutherford‐Appleton Laboratories, Didcot, UK). All samples were measured in disk‐shaped, fused silica banjo cells of 2 mm path length and thermostated at 25 °C. The scattering intensity, I(Q), of the various samples was determined as a function of the scattering vector, Q=(4π/λ)sin⁡(θ/2), where sin⁡(θ/2) is the scattering angle, by normalizing the relevant sample scattering to the sample’s transmission after subtraction of the scattering for the $D_{2}O$ solvent. When fitting the data, a background correction was included to account for any mismatch in the level of incoherent and inelastic scattering between the sample and the deuterated solvent. The levels of the fitted backgrounds were routinely inspected to ensure that they were of a physically reasonable magnitude. Model‐fitting of the SANS data for the systems analyzed was performed using the SasView package [48]. A variety of form factors were investigated, including triaxial ellipsoids of uniform scattering length density, and core–shell spherical, prolate, and oblate spheroids, each applied in conjunction with a hard‐sphere structure factor [49].

The weighted fit between the measured and calculated scattering ($I{\left(Q\right)}_{m}$ and $I{\left(Q\right)}_{c}$, respectively) was achieved through application of the Levenberg–Marquardt algorithm [50, 51], which the various model parameters adjusted to reduce the calculated $\chi^{2}$ value, obtained as

(3) $$\chi^{2}=\frac{\sum_{i=1}^{n}{\left\{I{\left(Q\right)}_{m}-I{\left(Q\right)}_{c}\right\}}^{2}}{w_{i}^{2}}$$

where n is the total number of measured data points, and $w_{i}$ is the uncertainty on each $I{\left(Q\right)}_{m}$.

## 3 Results

In this section, the results of the analysis will be summarized. The shape, size, and the internal structure of each mixed micelle will be described. Furthermore, volume estimations alongside with the density calculations will be presented. Finally, the neighbor analysis will be discussed.

### 3.1 Tyloxapol‐Induced Modulation of Micelle Shape and Size

Figure 2 presents the SASA, ellipticity, volume, and radius of gyration ($R_{g}$) for each system, characterizing the size and shape of the micelles as a function of Tyloxapol content. Summary statistics for these properties are provided in Table 2.

The TY39 and TY79 micelles exhibit the smallest SASA values (TY39: 47,717±24 Å^2^; TY79: 50,326±23 Å^2^). While the SASA values for the mixed micelles are broadly similar, there is a general trend of increasing SASA with decreasing Tyloxapol content, regardless of the degree of oligomerisation. Notably, all Tyloxapol‐containing micelles have lower SASA values than previously reported for the 100% Triton X‐100 micelles (~60,100±100 Å^2^) [40].

Average micelle volumes for each system are listed in Table 2. In the TY39 systems, increasing the proportion of Tyloxapol is associated with a general decrease in micelle volume. In contrast, in the TY79 systems, the 50% and 75% Tyloxapol micelles have smaller volumes than those with 25% and 100% Tyloxapol, which are similar in size. While the 25% and 50% Tyloxapol micelles from both TY39 and TY79 systems have comparable volumes, the volumetric differences between TY39 and TY79 micelles become more pronounced as the Tyloxapol content increases. These volumes are broadly consistent with the previously reported volume of the 100% Triton X‐100 micelle (73.8±0.5 nm^3^).

The $R_{g}$ values for all micelles are shown in Figure 2. Among the TY39 and TY79 systems, micelles with 25% and 75% Tyloxapol display larger radii of gyration than other compositions, with the exception of the 50% Tyloxapol 3 system, which is slightly larger than the 75% Tyloxapol 3 micelle. All Tyloxapol‐containing micelles have larger $R_{g}$ values than that of the previously reported 100% Triton X‐100 micelle. Furthermore, TY39‐TX100 mixed micelles consistently exhibit larger $R_{g}$ values than their TY79 counterparts at the same TX100 composition. Although, interestingly, the TY79 micelle has a slightly larger $R_{g}$ than the TY39 micelle.

To assess micelle shape, we computed the principal moments of inertia and corresponding ellipticity values (Figure 2). Table 2 provides average ellipticity values and corresponding shape classifications. Both TY39 and TY79 micelles are oblate, with average ellipticities of approximately 1.4. The TY39‐TX100 75% mixed micelle adopts a triaxial shape, while the remaining mixed micelles are prolate. All TY39‐TX100 micelles display eccentricities greater than 1.8, indicating they are considerably more aspherical than the TY39 and TY79 micelles, TY79‐TX100 micelles, or the 100% TX100 micelles.

The SANS measurements serve two purposes in this study: First, as an independent experimental cross‐check of the micelle size, aggregation number and overall (oblate) shape predicted by simulation for the pure Tyloxapol system; and second, as an independent estimate of core hydration, which we compare quantitatively with the simulated core water density in the following section. Because the SANS form factors were selected independently of the MD structures (see Section 2), agreement between the two methods on both an external (size/shape) and an internal (core hydration) property provides a genuine, independent validation of the simulation results.

**FIGURE 3:**
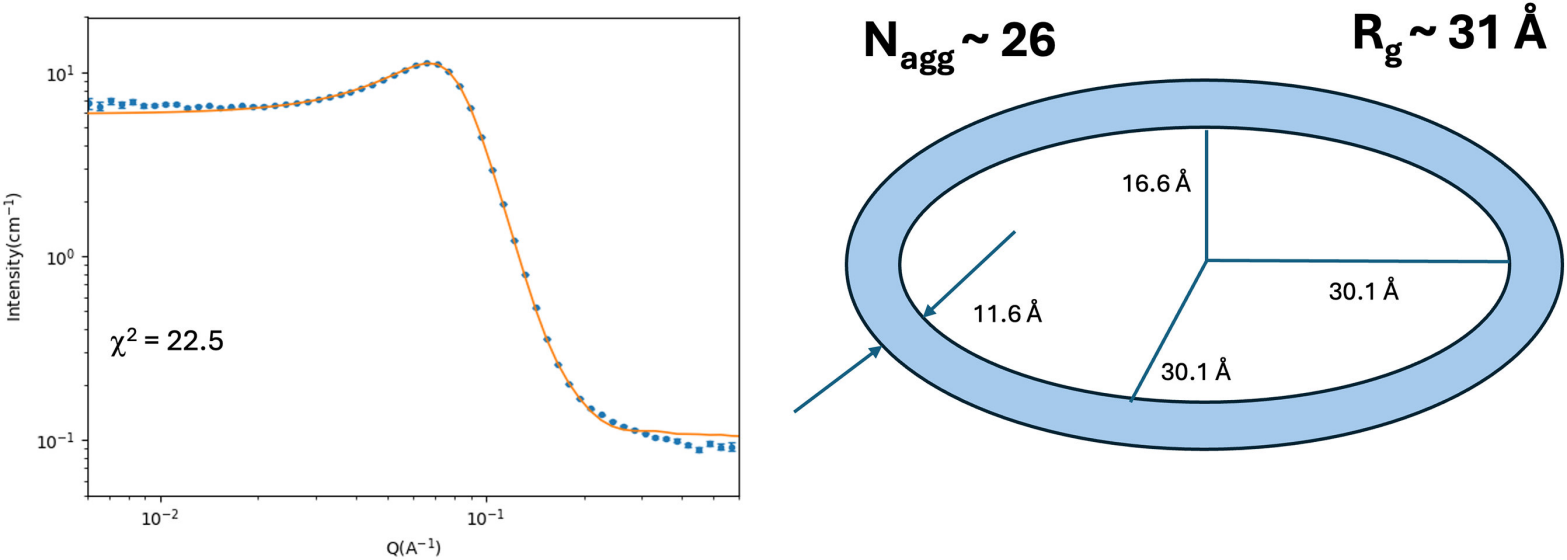
Neutron‐scattering profile and corresponding structural interpretation for Tyloxapol micelles. Left: scattering intensity as a function of the momentum transfer Q for Tyloxapol micelles in a 15% w/w Tyloxapol solution. The solid line shows the best‐fit model, obtained using a core–shell form factor combined with a hard‐sphere structure factor to capture inter‐micellar interactions. Right: schematic representation of the micelle dimensions extracted from the SANS analysis, illustrating the core–shell architecture identified from the fitted parameters.

We also performed SANS experiments to characterize Tyloxapol micelles in solution. In our experiments, the Tyloxapol molecules have 6–8 EO units in their hydrophilic head groups, and the number of monomers in the molecule is less than 6. In a 15% w/w solution, SANS analysis revealed an aggregation number of 26 Tyloxapol molecules (Figure 3), in good agreement with the 21 molecules included in our simulations. The experimental $R_{g}$ of approximately 31 Å and the oblate micelle shape are consistent with simulation findings (Table 2). The size of the micelles found from the SANS data for 1% w/w, 5% w/w, and 10% w/w solutions of Tyloxapol is constant (data provided in the Supporting Information).

### 3.2 Cavity Formation and Core Hydration in Tyloxapol‐Based Micelles

**FIGURE 4:**
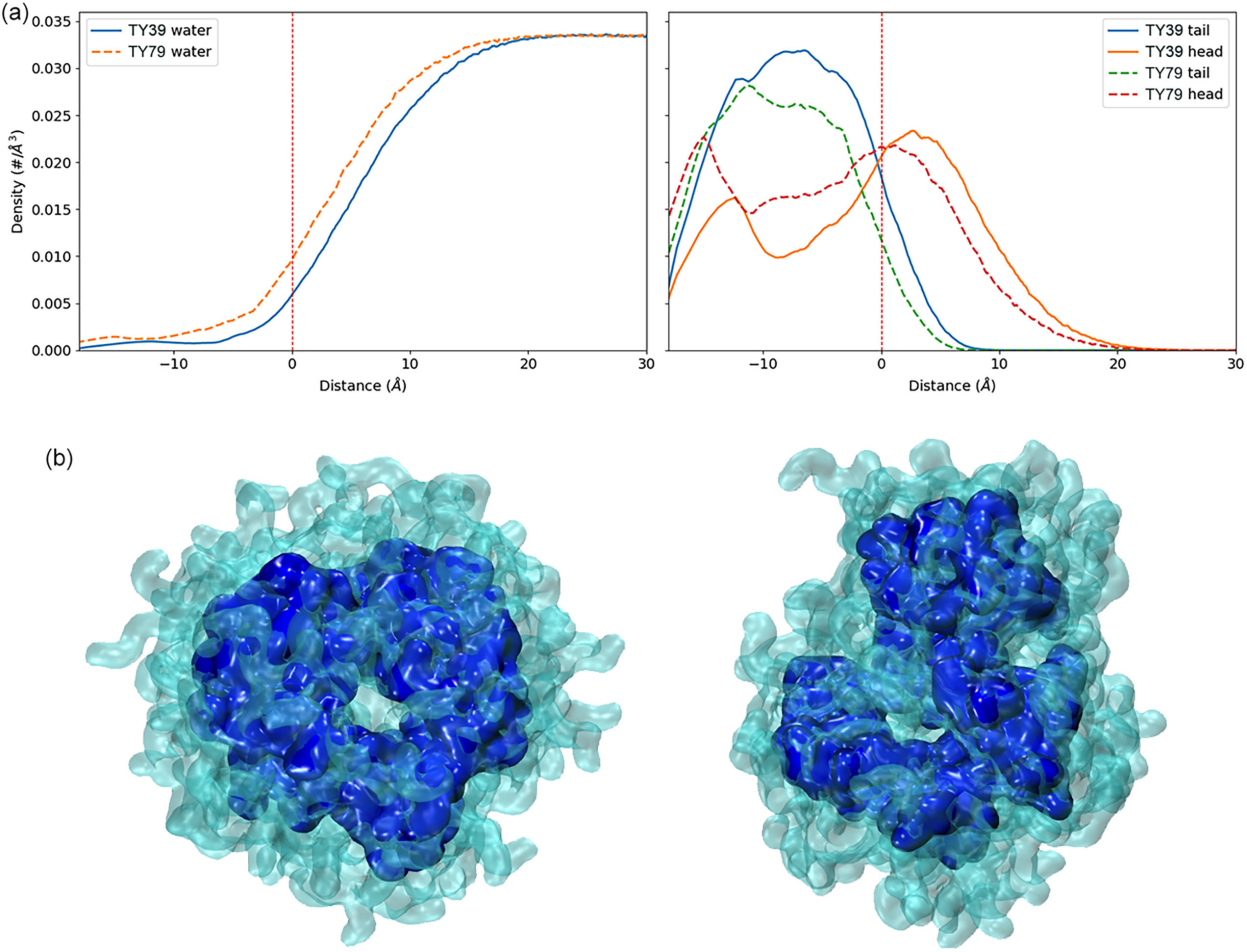
Structural organization and internal morphology of Tyloxapol micelles obtained from our molecular dynamics simulations. (a) Radial density profiles showing the distribution of water molecules (left) and the hydrophobic and hydrophilic components of the surfactants (right) for the TY39 and TY79 micelles. These profiles highlight differences in hydration and molecular packing arising from the distinct oligomer lengths. (b) Representative snapshots of the internal pore structures formed within the cores of the TY39 (left) and TY79 (right) micelles, illustrating how tyloxapol chain length influences core heterogeneity and internal void formation.

To characterize the internal structure of the micelles, we defined the hydrophobic–hydrophilic interface using the carbon atoms of the hydrophobic tails and the oxygen atoms closest to the tail regions of TX100 and Tyloxapol. The densities of hydrophobic tails, hydrophilic headgroups, and water molecules were calculated based on the positions of these atoms. Figure 4 shows the resulting density profiles for the TY39 and TY79 systems. As previously described, r=0 corresponds to any point on the surface of the micelle interface.

Figure 4a shows the water density in the TY39 and TY79 systems. In the bulk region (large r), the water density reaches ~0.033 Å^−3^, consistent with values reported using alternative methods [46, 52, 53]. Closer to the hydrophobic core–water interface, water density decreases due to steric exclusion by the large surfactant headgroups, reaching average values of $0.9\times 10^{-3}$ Å^−3^ for TY39 and $1.5\times 10^{-3}$ Å^−3^ for TY79. These values indicate the presence of a small amount of water within the micelle cores. Table 3 reports the total number of water molecules and the number of water molecules per unit volume in each micelle. SANS analysis of a 15% w/w Tyloxapol solution yielded a water density of 3.3 nm^−3^ within the core of the micelle, which is in good agreement with the simulation result for the TY79 system ($n_{w}=2.8$ nm^−3^).

**TABLE 3:** Key structural and compositional metrics extracted from the molecular dynamics simulations of Triton X‐100 and tyloxapol micelles. The table reports the number density of water molecules within the micelle interior ($n_{w}$), the total number of encapsulated water molecules ($N_{H_{2}O}$), the number of aromatic π−π stacking interactions between surfactant hydrophobic tails ($N_{\pi \text{−}\pi }$), the number of PEG monomers from the surfactant headgroups located within the micelle core ($N_{\text{PEG}}$). Together, these metrics quantify differences in hydration, packing, and internal organization across the mixed‐ and single‐component micellar systems.

System	$n_{w}$, #/nm^3^	$N_{H_{2}O}$	$N_{\pi \text{−}\pi }$	$N_{\text{PEG}}$
TY39	1.7	137±15	6±3	12±2
TY39‐TX100 75	3.8	298±44	5±2	49±2
TY39‐TX100 50	2.7	211±31	7±3	21±4
TY39‐TX100 25	2.9	259±45	3±3	27±5
TY79	2.8	255±22	5±2	14±1
TY79‐TX100 75	2.0	181±21	2±2	23±3
TY79‐TX100 50	1.9	156±40	8±3	13±3
TY79‐TX100 25	3.4	261±51	3±2	26±5

Figure 4b presents the density profiles of the hydrophobic tails and hydrophilic headgroups for the TY39 (trimer) and TY79 (heptamer) systems. In both cases, the density of the hydrophobic tails exceeds that of the headgroups throughout the micelle core. Nevertheless, a notable peak in the density of headgroup atoms at r< −10 Å indicates that portions of the headgroups penetrate into the micelle core. In TY39 micelles, this results in a single internal pore, while in TY79 micelles, multiple cavities are formed (Figure 4b). These regions are typically where the trapped water is located.

We note that internal cavities and headgroup/water penetration of this kind recur across all Tyloxapol‐containing compositions examined (TY39, TY79, and the corresponding mixed systems; Figure S6), with the degree of internal porosity decreasing systematically as Triton X‐100 content increases. Because each composition was built from an independently packed initial configuration, this recurring, composition‐dependent pattern across multiple systems provides some support that the cavities reflect a genuine structural feature rather than an artifact specific to a single starting configuration; nonetheless, we did not carry out independent replicate assemblies at fixed composition, and we identify this as the clearest way to confirm that the inhomogeneities persist (see Section 4).

Similar density analyses were conducted for the mixed systems and are shown in Figure S6. In the TY79‐TX100 75 and TY39‐TX100 75 micelles (Figure S6a,b), a significant amount of water is present in the micelle core, accompanied by a high density of hydrophilic headgroup atoms. As in the TY39 and TY79 systems, these mixed micelles contain internal cavities within the hydrophobic region, where headgroups and water molecules are located. As the number of TX100 molecules increases, the micelle core becomes more compact and less porous, which is reflected in the lower internal headgroup densities in Figure S6c,e, f. However, the TY79‐TX100 50 system still displays some internal gaps, albeit smaller than those in systems with higher Tyloxapol content.

To further investigate the molecular interactions within the hydrophobic core, we analyzed *π*–*π* stacking interactions using the RSA tool in the PySoftK package. Table 3 lists the average number of *π*–*π* interactions ($N_{\pi –\pi }$) per system. A negative correlation is observed between the amount of trapped water in the micelle core and the number of stacked aromatic rings between surfactant tails. Notably, the 100% and 50% Tyloxapol systems exhibit the highest number of *π*–*π* interactions and the lowest water content in the core. These results suggest that the formation of internal pores and water retention may be closely linked to the molecular structure and interaction patterns of the constituent surfactant molecules.

### 3.3 No Preferential Association Observed Between Tyloxapol and Triton X‐100 Molecules

**FIGURE 5:**
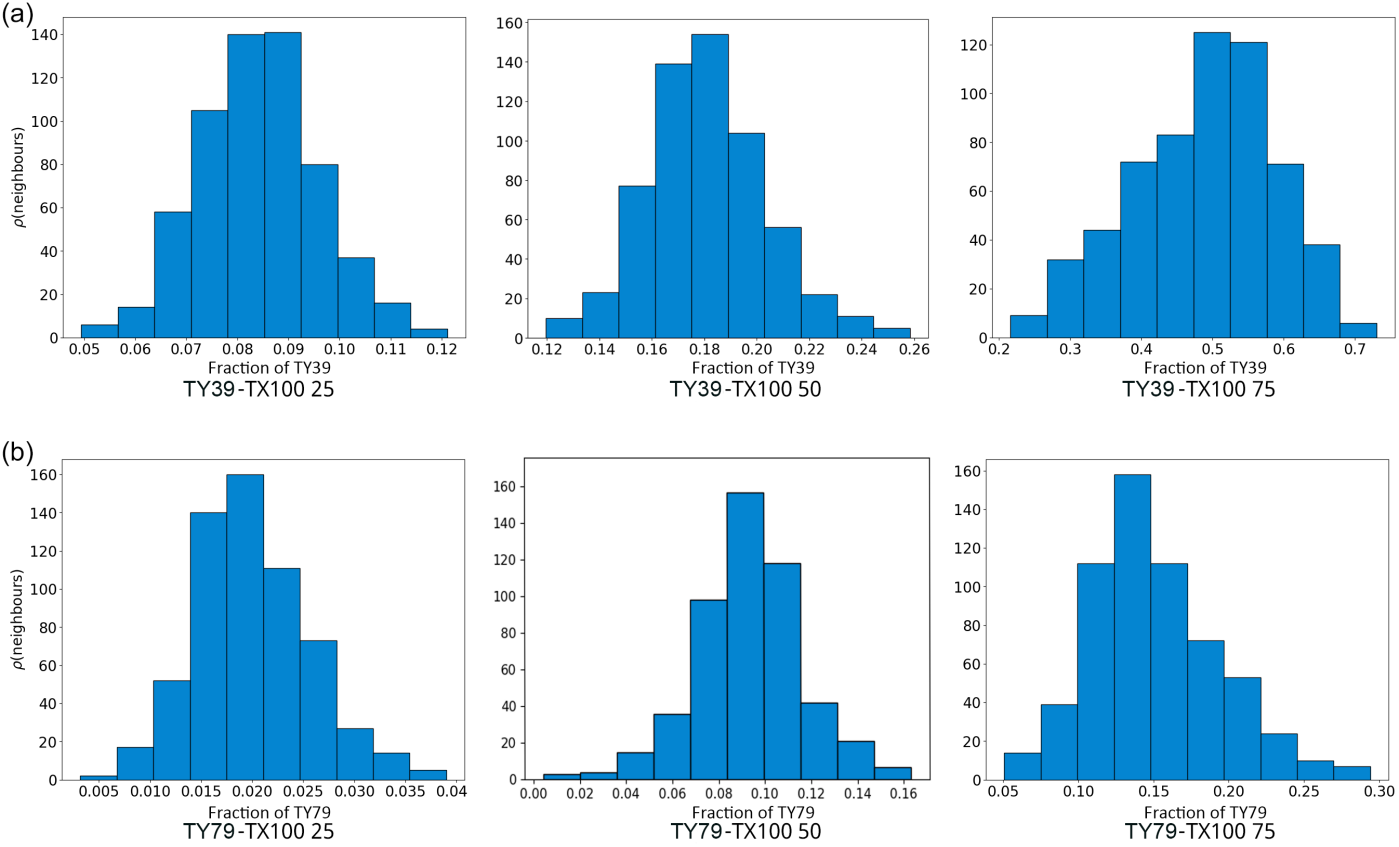
Local organzation of Tyloxapol around Triton X‐100 surfactants as determined from our molecular dynamics simulations. Shown is the fraction of Tyloxapol (a) trimers and (b) heptamers located in the local environment of TX100 molecules, highlighting how oligomer length influences surfactant–surfactant mixing and spatial association within the micelles.

To evaluate the local environment of each surfactant type within the micelles, a neighbor analysis is performed. Figure 5a shows the distribution of TY39 molecules surrounding the TX100 molecules throughout the simulation trajectory. The average TY39 neighbor fractions around TX100 molecules in the TY39‐TX100 25%, 50%, and 75% systems are 0.09, 0.18, and 0.48, respectively. These values closely align with the actual fraction of Tyloxapol molecules present within the micelles: ∼0.10 (TY39‐TX100 25), 0.25 (TY39‐TX100 50), and 0.50 (TY39‐TX100 75). The similarity between the compositional and neighbor fractions indicates a lack of preferential association or selectivity between Tyloxapol trimers (TY39) and Triton X‐100 molecules.

A similar neighbor analysis is conducted for the TY79‐TX100 mixed micelles and is shown in Figure 5b. The TY79 neighbor fractions surrounding TX100 molecules in the 25% and 50% systems are 0.03 and 0.10, respectively, while the corresponding fractions of TY79 molecules within the micelles are 0.04 and 0.13. For the TY79‐TX100 75 system, the average TY79 neighbor fraction is 0.20, compared to an actual micelle composition of 0.30 TY79. While there is a slight discrepancy between the neighbor and compositional ratios in this system, it does not necessarily imply molecular selectivity. Due to the larger size and reduced mobility of the Tyloxapol heptamers (TY79), the local neighborhood around each molecule remained relatively stable throughout the simulation.

### 3.4 Micelle Composition Dictates Tyloxapol Conformational Landscapes

**FIGURE 6:**
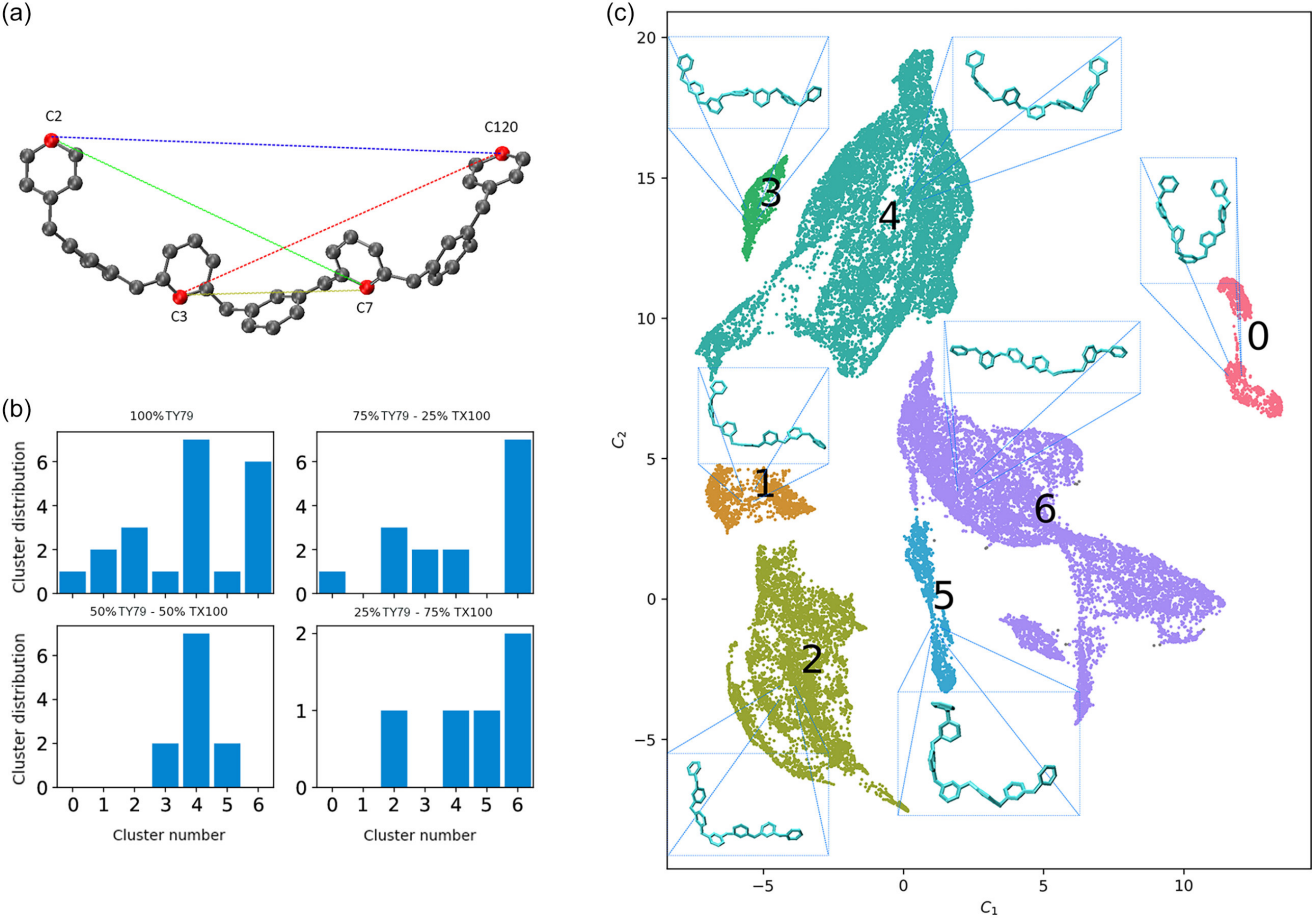
Machine‐learning characterization of Tyloxapol conformations derived from our molecular dynamics simulations. (a) Four intramolecular distances (dashed lines) defined between the highlighted red atoms were used as structural descriptors to represent the conformational state of TY79 molecules. (b) Distribution of the identified TY79 conformational states across the different simulations, illustrating how micelle composition influences the conformations sampled. (c) Two‐dimensional embedding of the distance‐based feature space, with clustering revealing the distinct conformational families adopted by TY79 within the micelle environment.

To characterize the conformations of TY79 molecules within the micelles, four intramolecular distances were selected between specific carbon atoms (C3‐C7, C2‐C7, C3‐C120, and C2‐C120) to represent the structural state of each Tyloxapol molecule (Figure 6a). These distance vectors were used as input for dimensionality reduction using UMAP, with the n_neighbors parameter set to 20, resulting in a two‐dimensional embedded space.

Subsequently, clustering is performed using HDBSCAN to identify distinct conformational states. The clustering algorithm is parameterized with min_cluster_size set to 1000 and min_samples set to 16. The large value for min_cluster_size is chosen due to the dense population of points in the embedded space, while the smaller min_samples value allowed the algorithm to classify as few points as noise as possible, thereby capturing a wider range of conformational variation.

Figure 6c shows the resulting clusters, with representative molecular conformations visualized for each cluster. The dataset included TY79 molecules from all simulations containing Tyloxapol heptamers, including both the TY79 systems and TY79‐Triton X‐100 mixed systems. The distribution of conformations varies across systems, with some conformers found only in specific micellar environments. For example, the L‐shaped conformation represented by cluster “2” appears exclusively in the TY79 system, while the U‐shaped conformation of cluster “0” is observed in both the TY79 and TY79‐TX100 75 systems, but not in others. The distribution of conformations across systems is shown in Figure 6b.

## 4 Conclusions

In this study, we investigated the structural characteristics and internal organization of mixed micelles composed of Triton X‐100 and its oligomeric analogue, Tyloxapol (in trimeric and heptameric forms), using all‐atom MD simulations. Our results show that the incorporation of Tyloxapol significantly alters the size, shape, and internal morphology of the micelles.

The TY39 and TY79 micelles exhibited smaller SASAs and more compact structures compared to 100% Triton X‐100 micelles, with both TY39 and TY79 systems adopting oblate geometries. Mixed micelles displayed a broader range of morphologies, depending on the Tyloxapol content and oligomer length. In particular, TY39‐TX100 mixtures tended toward more elongated (prolate) or triaxial shapes, especially at higher TX100 concentrations.

Analysis of internal micelle structure revealed the presence of water‐filled cavities and the penetration of hydrophilic headgroups into the micelle core, particularly in systems rich in Tyloxapol. This phenomenon is more pronounced in TY79‐containing micelles, suggesting that longer oligomers promote a more heterogeneous internal environment. The presence of these cavities is correlated with a reduction in *π*–*π* stacking interactions between aromatic moieties on the surfactant tails, indicating a complex interplay between molecular packing and core hydration. Recent work by Cezar et al. has shown, using a combination of scattering experiments and data‐driven simulations, that Triton X‐100 micelles can exhibit a variety of internal structures [54], consistent with our observations for both the TY39 and TY79 micelles and the mixed systems.

Neighborhood analysis revealed no strong preference for self‐association or cross‐association between Tyloxapol and Triton X‐100, indicating random mixing of the surfactants within the micelle. Furthermore, unsupervised machine learning applied to intramolecular geometries of TY79 molecules identified distinct conformational states, some of which were unique to specific system compositions.

We note that, as with essentially all atomistic simulations of micelles of this size, our approach relies on a preassembled, approximately spherical starting configuration rather than unbiased self‐assembly, which remains computationally inaccessible at this level of resolution. This choice imposes an initial globular geometry and packing density that may not emerge spontaneously, and some internal structural features, in particular the cavities discussed above, could in principle retain a residual dependence on the starting configuration. We mitigated this by simulating each system for substantially longer than the time required for global size and shape descriptors to reach a stable plateau (Figures S1–S4), by applying an identical preassembly protocol to every composition so that any residual bias affects all systems comparably, and, for the pure Tyloxapol system, by independently validating the simulated size and shape against SANS measurements. Nonetheless, confirming that the internal cavities and hydration patterns reported here are insensitive to the initial packing will require independent replicate assemblies, which we identify as a priority for future work.

Together, these findings provide new mechanistic insight into how oligomerisation and surfactant mixing influence micelle structure and stability. To our knowledge, this is the first computational study to explore mixed micelles formed from a monomeric surfactant and its oligomeric counterpart. The results presented here have implications for the design of micellar systems for drug delivery and nanotechnological applications, where precise control over internal micellar architecture is essential.

## Author Contributions

H.I., D.J.B., M.J.L., and C.D.L. designed the research; H.I., D.J.B., and M.J.L. performed the research; H.I. and D.J.B. analyzed the data; H.I. and C.D.L. wrote the initial draft of the manuscript; H.I., D.J.B., M.J.L., and C.D.L. edited the manuscript.

## Funding

This study was supported by the Engineering and Physical Sciences Research Council (EP/T022213/1, EP/W032260/1, and EP/P020194/1), the National Science Foundation (DMR‐0520547), Horizon 2020 Framework Programme (654000), and the Higher Education and Science Committee of the Republic of Armenia (N 24RL‐1C009).

## Conflicts of Interest

The authors declare no conflicts of interest.

## Supporting information

Supplementary Material

## Data Availability

The data that support the findings of this study are available from the corresponding author upon reasonable request.

## References

[1] M. J. L. Castro , C. Ojeda , and A. F. Cirelli , Surfactants in Agriculture, in Green Materials for Energy, Products and Depollution, Environmental Chemistry for a Sustainable World, ed. E. Lichtfouse , J. Shwarzbauer and D. Robert , vol 3 (Springer, 2013), 10.1007/978-94-007-6836-9_7.

[2] C. C. West and J. H. Harwell , “Surfactants and Subsurface Remediation,” Environmental Science & Technology 26 (1992): 2324–2330.

[3] P. J. Stevens , “Organosilicone Surfactants as Adjuvants for Agrochemicals,” Pesticide Science 38 (1993): 103–122.

[4] B. S. Sekhon , “Surfactants: Pharmaceutical and Medicinal Aspects,” Journal of Pharmaceutical Technology, Research and Management 1 (2013): 43–68.

[5] A. Laschewsky , K. Lunkenheimer , R. H. Rakotoaly , and L. Wattebled , “Spacer Effects in Dimeric Cationic Surfactants,” Colloid and Polymer Science 283 (2005): 469–479.

[6] R. Zana , “Dimeric and Oligomeric Surfactants. Behavior at Interfaces and in Aqueous Solution: A Review,” Advances in Colloid and Interface Science 97 (2002): 205–253. 12027021 10.1016/s0001-8686(01)00069-0

[7] J. Chen , M. Qiao , N. Gao , et al., “Cationic Oligomeric Surfactants as Novel Air Entraining Agents for Concrete,” Colloids and Surfaces a: Physicochemical and Engineering Aspects 538 (2018): 686–693.

[8] M. In , V. Bec , O. Aguerre‐Chariol , and R. Zana , “Quaternary Ammonium Bromide Surfactant Oligomers in Aqueous Solution: Self‐Association and Microstructure,” Langmuir 16 (2000): 141–148.

[9] C. A. Bunton , L. B. Robinson , J. Schaak , and M. F. Stam , “Catalysis of Nucleophilic Substitutions by Micelles of Dicationic Detergents,” The Journal of Organic Chemistry 36 (1971): 2346–2350.

[10] C. Zhou , F. Wang , H. Chen , et al., “Selective Antimicrobial Activities and Action Mechanism of Micelles Self‐Assembled by Cationic Oligomeric Surfactants,” ACS Applied Materials & Interfaces 8 (2016): 4242–4249. 26820390 10.1021/acsami.5b12688

[11] N. Dharaiya , V. K. Aswal , and P. Bahadur , “Characterization of Triton X‐100 and Its Oligomer (Tyloxapol) Micelles Vis‐à‐Vis Solubilization of Bisphenol a by Spectral and Scattering Techniques,” Colloids and Surfaces a: Physicochemical and Engineering Aspects 470 (2015): 230–239.

[12] T. Dam , J. Engberts , J. Karthäuser , S. Karaborni , and N. van Os , “Synthesis, Surface Properties and Oil Solubilisation Capacity of Cationic Gemini Surfactants,” Colloids and Surfaces a: Physicochemical and Engineering Aspects 118 (1996): 41–49.

[13] P. K. Maiti , Y. Lansac , M. A. Glaser , N. A. Clark , and Y. Rouault , “Self‐Assembly in Surfactant Oligomers: A Coarse‐Grained Description Through Molecular Dynamics Simulations,” Langmuir 18 (2002): 1908–1918.

[14] H. Wu , J. Xu , X. He , Y. Zhao , and H. Wen , “Mesoscopic Simulation of Self‐Assembly in Surfactant Oligomers by Dissipative Particle Dynamics,” Colloids and Surfaces a: Physicochemical and Engineering Aspects 290 (2006): 239–246.

[15] P. Wang , S. Pei , M. Wang , Y. Yan , X. Sun , and J. Zhang , “Coarse‐Grained Molecular Dynamics Study on the Self‐Assembly of Gemini Surfactants: The Effect of Spacer Length,” Physical Chemistry Chemical Physics 19 (2017): 4462–4468. 28120957 10.1039/c6cp07690d

[16] J. F. Scamehorn , ed. Phenomena in Mixed Surfactant Systems, ACS Symposium Series 311 (American Chemical Society, 1986), 10.1021/bk-1986-0311.

[17] Y. Moroi , Mixed Micelle Formation (Springer, 1992), 10.1007/978-1-4899-0700-4_10.

[18] P. M. Holland and D. N. Rubingh , eds. Mixed Surfactant Systems, ACS Symposium Series 501 (American Chemical Society, 1992), 10.1021/bk-1992-0501.

[19] M. E. Haque , A. R. Das , A. K. Rakshit , and S. P. Moulik , “Properties of Mixed Micelles of Binary Surfactant Combinations,” Langmuir 12 (1996): 4084–4089.

[20] A. Khan and E. F. Marques , “Synergism and Polymorphism in Mixed Surfactant Systems,” Current Opinion in Colloid & Interface Science 4 (1999): 402–410.

[21] D. P. Acharya and H. Kunieda , “Wormlike Micelles in Mixed Surfactant Solutions,” Advances in Colloid and Interface Science 123–126 (2006): 401–413. 10.1016/j.cis.2006.05.024 16860768

[22] G. Sugihara , S. Nagadome , S. W. Oh , and J. S. Ko , “A Review of Recent Studies on Aqueous Binary Mixed Surfactant Systems,” Journal of Oleo Science 57 (2008): 61–92. 18198464 10.5650/jos.57.61

[23] Y. Mrestani , L. Behbood , H. artl , A. Neubert , and H. R.H., “Microemulsion and Mixed Micelle for Oral Administration as New Drug Formulations for Highly Hydrophilic Drugs,” European Journal of Pharmaceutics and Biopharmaceutics 74 (2010): 219–222. 19932178 10.1016/j.ejpb.2009.11.009

[24] V. Alakhov , E. Klinski , S. Li , et al., “Block Copolymer‐Based Formulation of Doxorubicin. From Cell Screen to Clinical Trials,” Colloids and Surfaces B: Biointerfaces 16 (1999): 113–134.

[25] H. S. Yoo and T. G. Park , “Folate Receptor Targeted Biodegradable Polymeric Doxorubicin Micelles,” Journal of Controlled Release 96 (2004): 273–283. 15081218 10.1016/j.jconrel.2004.02.003

[26] Z. G. Gao , H. D. Fain , and N. Rapoport , “Controlled and Targeted Tumor Chemotherapy by Micellar‐Encapsulated Drug and Ultrasound,” Journal of Controlled Release : Official Journal of the Controlled Release Society 102 (2005): 203–222. 15653146 10.1016/j.jconrel.2004.09.021

[27] X. L. Wu , J. H. Kim , H. Koo , et al., “Tumor‐Targeting Peptide Conjugated pH‐Responsive Micelles as a Potential Drug Carrier for Cancer Therapy,” Bioconjugate Chemistry 21 (2010): 208–213. 20073455 10.1021/bc9005283

[28] S. Mehta and N. Jindal , “Mixed Micelles of LecithIn‐Tyloxapol as Pharmaceutical Nanocarriers for Anti‐Tubercular Drug Delivery,” Colloids and Surfaces B: Biointerfaces 110 (2013): 419–425. 23751420 10.1016/j.colsurfb.2013.05.015

[29] D. C. Turner , F. Yin , J. T. Kindt , and H. Zhang , “Molecular Dynamics Simulations of Glycocholate‐Oleic Acid Mixed Micelle Assembly,” Langmuir 26 (2010): 4687–4692. 20112949 10.1021/la903573m

[30] S. Storm , S. Jakobtorweihen , and I. Smirnova , “Solubilization in Mixed Micelles Studied by Molecular Dynamics Simulations and COSMOmic,” Journal of Physical Chemistry B 118 (2014): 3593–3604. 24533791 10.1021/jp410636w

[31] W. Wu , Z. Zou , S. Yang , et al., “Coarse‐Grained Molecular Dynamic and Experimental Studies on Self‐Assembly Behavior of Nonionic F127/HS15 Mixed Micellar Systems,” Langmuir 36 (2020): 2082–2092. 32088962 10.1021/acs.langmuir.9b03936

[32] H. Bekker , H. Berendsen , E. Dijkstra , et al., “A Parallel Computer for Molecular Dynamics Simulations,” Physics Computing 92 (1993): 252–256.

[33] H. Berendsen , D. van der Spoel , and R. van Drunen , “GROMACS: A Message‐Passing Parallel Molecular Dynamics Implementation,” Computer Physics Communications 91 (1995): 43–56.

[34] M. J. Abraham , T. Murtola , R. Schulz , et al., “GROMACS: High Performance Molecular Simulations Through Multi‐Level Parallelism From Laptops to Supercomputers,” SoftwareX 1‐2 (2015): 19–25.

[35] B. R. Brooks , R. E. Bruccoleri , B. D. Olafson , D. J. States , S. Swaminathan , and M. Karplus , “CHARMM: A Program for Macromolecular Energy, Minimization, and Dynamics Calculations,” Journal of Computational Chemistry 4 (1983): 187–217.

[36] J. Huang and A. D. Mackerell , “CHARMM36 All‐Atom Additive Protein Force Field: Validation Based on Comparison to NMR Data,” Journal of Computational Chemistry 34 (2013): 2135–2145. 23832629 10.1002/jcc.23354 PMC3800559

[37] J. Huang , S. Rauscher , G. Nawrocki , et al., “CHARMM36m: An Improved Force Field for Folded and Intrinsically Disordered Proteins,” Nature Methods 14 (2017): 71–73. 27819658 10.1038/nmeth.4067 PMC5199616

[38] D. Yordanova , I. Smirnova , and S. Jakobtorweihen , “Molecular Modeling of Triton X Micelles: Force Field Parameters, Self‐Assembly, and Partition Equilibria,” Journal of Chemical Theory and Computation 11 (2015): 2329–2340. 26574428 10.1021/acs.jctc.5b00026

[39] K. Vanommeslaeghe , E. Hatcher , C. Acharya , et al., “CHARMM General Force Field: A Force Field for Drug‐Like Molecules Compatible With the CHARMM All‐Atom Additive Biological Force Fields,” Journal of Computational Chemistry 31 (2010): 671–690. 19575467 10.1002/jcc.21367 PMC2888302

[40] H. Ishkhanyan , N. H. Rhys , D. J. Barlow , M. J. Lawrence , and C. D. Lorenz , “Impact of Drug Aggregation on the Structural and Dynamic Properties of Triton X‐100 Micelles,” Nanoscale 14 (2022): 5392–5403. 35319029 10.1039/d1nr07936k

[41] L. Martínez , R. Andrade , E. G. Birgin , and J. M. Martínez , “PACKMOL: A Package for Building Initial Configurations for Molecular Dynamics Simulations,” Journal of Computational Chemistry 30 (2009): 2157–2164. 19229944 10.1002/jcc.21224

[42] B. Hess , H. Bekker , H. J. C. Berendsen , and J. G. E. M. Fraaije , “LINCS: A Linear Constraint Solver for Molecular Simulations,” Journal of Computational Chemistry 18 (1997): 1463–1472.

[43] H. Ishkhanyan , A. Santana‐Bonilla , and C. D. Lorenz , “PUCHIK: A Python Package to Analyze Molecular Dynamics Simulations of Aspherical Nanoparticles,” Journal of Chemical Information and Modeling 65 (2025): 1694–1701. 39928985 10.1021/acs.jcim.4c02128 PMC11863366

[44] L. McInnes , J. Healy , N. Saul , and L. Großberger , “UMAP: Uniform Manifold Approximation and Projection,” Journal of Open Source Software 3 (2018): 861.

[45] L. McInnes , J. Healy , and S. Astels , “hdbscan: Hierarchical Density Based Clustering,” Journal of Open Source Software 2 (2017): 205.

[46] R. M. Ziolek , P. Smith , D. L. Pink , C. A. Dreiss , and C. D. Lorenz , “Unsupervised Learning Unravels the Structure of Four‐Arm and Linear Block Copolymer Micelles,” Macromolecules 54 (2021): 3755–3768.

[47] R. M. Ziolek , A. Santana‐Bonilla , R. López‐Ríos de Castro , R. Kühn , M. Green , and C. D. Lorenz , “Conformational Heterogeneity and Interchain Percolation Revealed in an Amorphous Conjugated Polymer,” ACS Nano 16 (2022): 14432–14442. 36103148 10.1021/acsnano.2c04794 PMC9527807

[48] M. Doucet , J. H. Cho , G. Alina , et al., SasView version 4.2 (2018), 10.5281/zenodo.1412040.

[49] J. K. Percus and G. J. Yevick , “Analysis of Classical Statistical Mechanics by Means of Collective Coordinates,” The Physical Review 110 (1958): 1–13.

[50] O. R. Pal , V. G. Gaikar , J. V. Joshi , P. S. Goyal , and V. K. Aswal , “Small‐Angle Neutron Scattering Studies of Mixed Cetyl Trimethylammonium Bromidebutyl Benzene Sulfonate Solutions,” Langmuir 18 (2002): 6764–6768.

[51] K. V. Padalkar , V. G. Gaikar , and V. K. Aswal , “Characterization of Mixed Micelles of Sodium Cumene Sulfonate With Sodium Dodecyl Sulfate and Cetyl Trimethylammonium Bromide by SANS, FTIR Spectroscopy and NMR Spectroscopy,” Journal of Molecular Liquids 144 (2009): 40–49.

[52] D. T. Allen , Y. Saaka , M. J. Lawrence , and C. D. Lorenz , “Atomistic Description of the Solubilisation of Testosterone Propionate in a Sodium Dodecyl Sulfate Micelle,” Journal of Physical Chemistry B 118 (2014): 13192–13201. 25343221 10.1021/jp508488c

[53] D. T. Allen and C. D. Lorenz , “A Novel Method for Constructing Continuous Intrinsic Surfaces of Nanoparticles,” Journal of Molecular Modeling 23 (2017): 1–11. 28674837 10.1007/s00894-017-3378-9 PMC5495850

[54] H. M. Cezar , V. A. Bj , S. Prévost , R. Lund , and M. Cascella , “Beyond Core‐Shell Micellar Structures: Complex Structures in Simple Surfactants,” Small Structures 6 (2025): 2400553.

[55] e Research King’s Computational Research , Engineering and Technology Environment (CREATE), 2025, 10.18742/rnvf-m076.

