# Supplementary material for: Oligomerisation Drives Internal Cavity Formation and Conformational Heterogeneity in Mixed Triton X‐100/Tyloxapol Micelles
Source: Small Sci. 2026 Aug 22;6(8):e70376. doi: 10.1002/smsc.70376 (PMC13516858; doi:10.1002/smsc.70376)
Supplement: Supplementary file 1 [file SMSC-6-e70376-s001.pdf]

Supporting information for "Oligomerisation Drives Internal Cavity Formation and Conformational Heterogeneity in Mixed Triton X-100/Tyloxapol Micelles"

Hrachya Ishkhanyan,[†,‡] David J. Barlow,[¶] M. Jayne Lawrence,[¶,§] and Christian D. Lorenz[*,‖,†]

†*Computational Molecular Engineering Lab, Institute for Informatics and Automation Problems of the National Academy of Sciences of the Republic of Armenia, 1, P. Sevak str. Yerevan, Republic of Armenia*

‡*Biological & Soft Matter Research Group, Department of Physics, Faculty of Natural, Mathematical & Engineering Sciences, King's College London, London, United Kingdom*

¶*Division of Pharmacy and Optometry, School of Health Sciences, Faculty of Biology, Medicine and Health, University of Manchester, Stopford Building, Oxford Road, Manchester, United Kingdom*

§*The University of Manchester at Harwell, Diamond Light Source, Harwell Campus, Didcot, Oxfordshire, OX11 0DE, UK*

‖*Department of Engineering, Faculty of Natural, Mathematical & Engineering Sciences, King's College London, London, United Kingdom*

E-mail: chris.lorenz@kcl.ac.uk

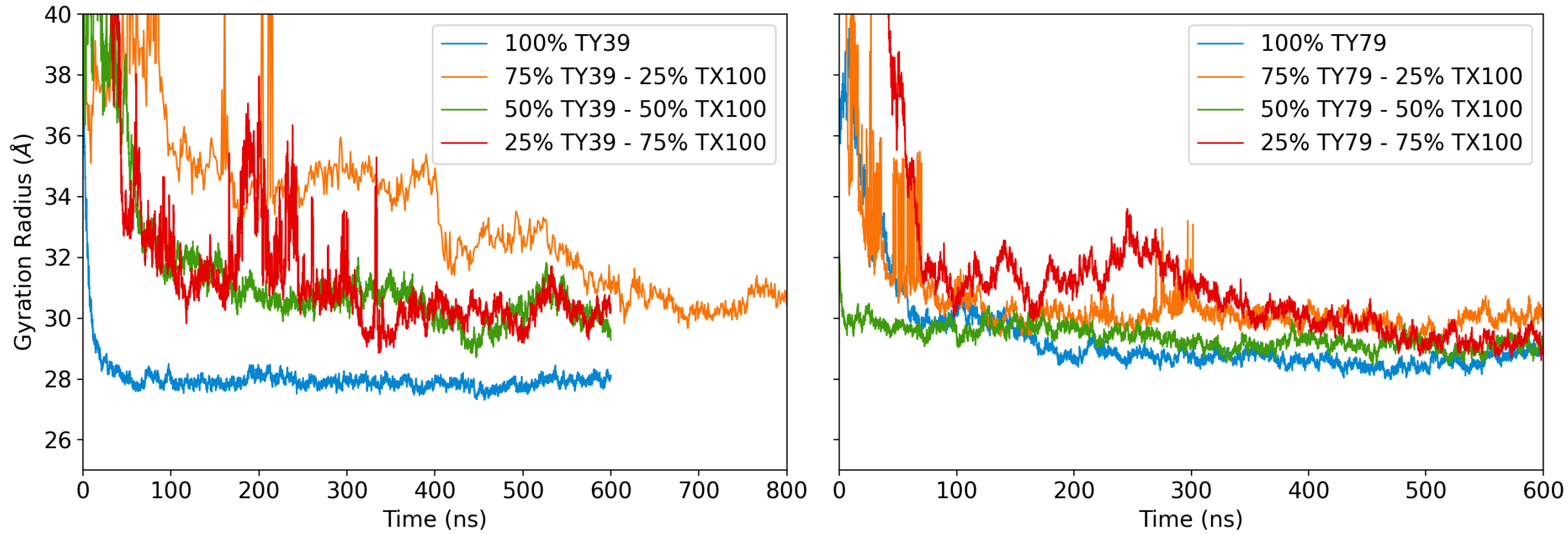

Figure S1: Radius of gyration of Tyloxapol trimer (left) and heptamer (right) based micelles as a function of time.

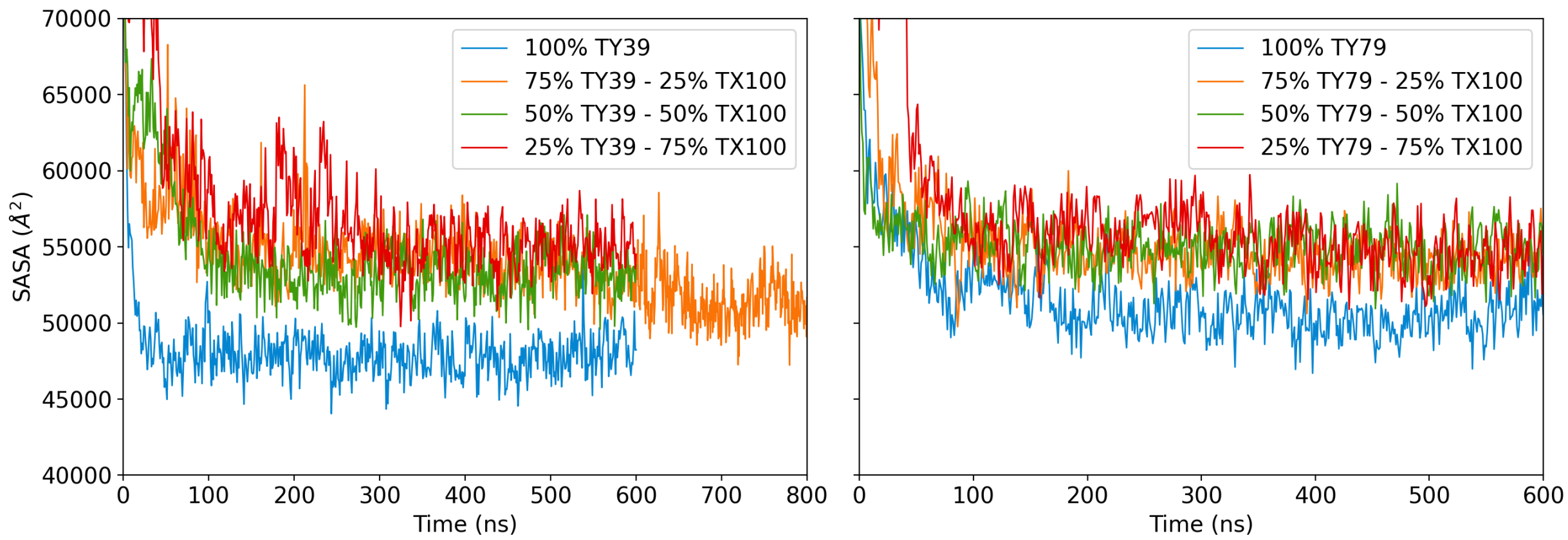

Figure S2: Solvent accessible surface area (SASA) of Tyloxapol trimer (left) and heptamer (right) based micelles as a function of time.

Results of SANS experiments of Tyloxapol solutions

Figures S7, S8 and S9 show the SANS scattering profile and schematics displaying the size and shape of the micelles found in the 1% w/w, 5% w/w and 10% w/w Tyloxapol solutions, respectively. In all solutions, the micelles are found to be oblate. The ratio of the major and minor axes of the micelles is 2.6 in the 1% w/w solution and then decreases to 1.8 for the three larger concentrations. The radius of gyration, R_g, found for all concentrations studied is 31 Å.

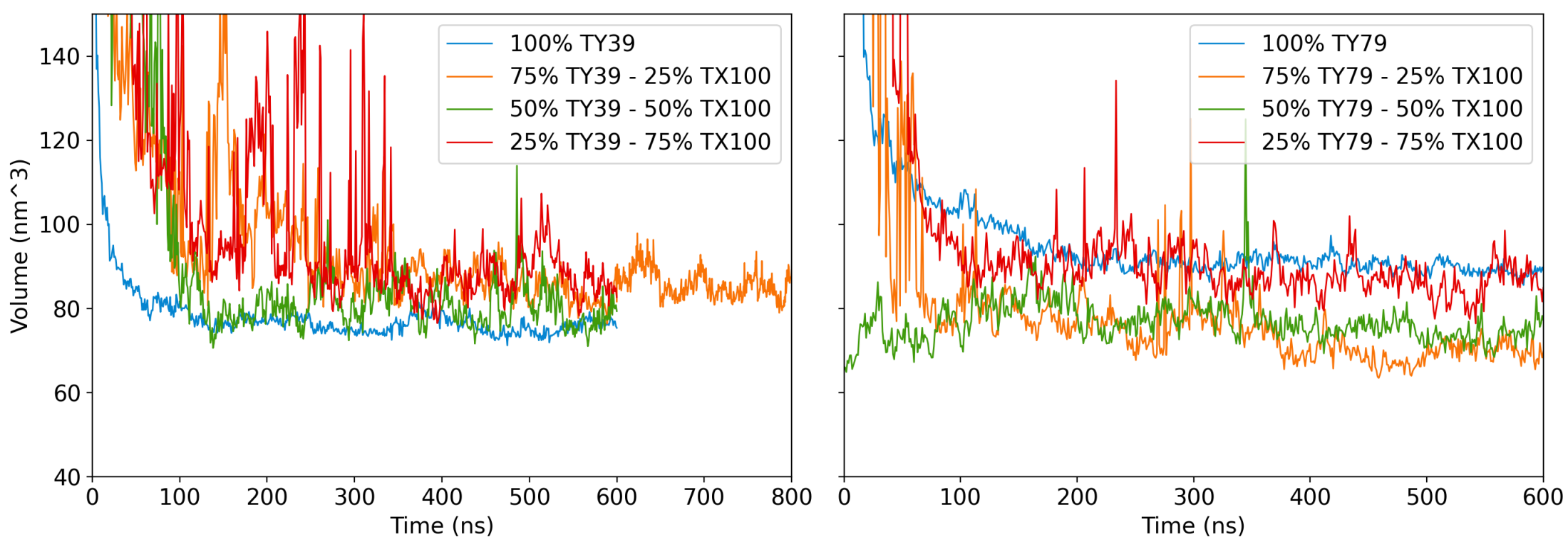

Figure S3: Volumes of Tyl3 (left) and Tyl7 (right) micelles as a function of time.

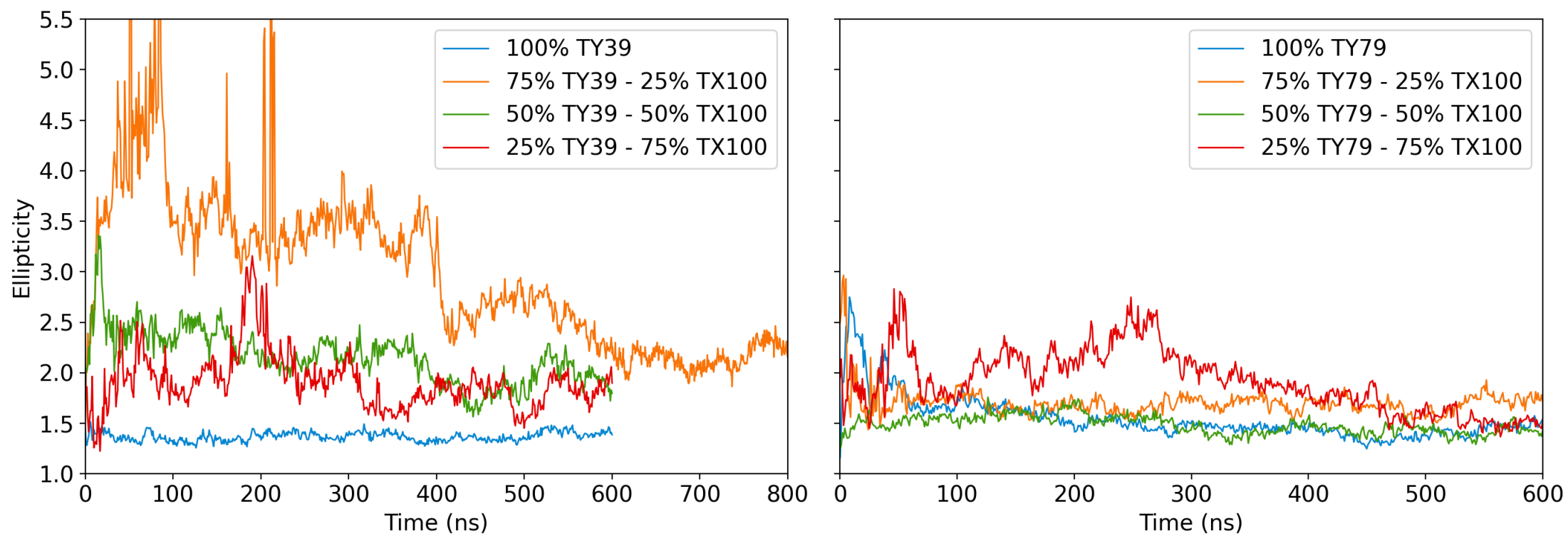

Figure S4: Ellipticity of Tyloxapol trimer (left) and heptamer (right) based micelles as a function of time.

Table S1 reports the core-shell interface surface area, the volume of the core and of the whole micelle that was determined from the SANS data for the different concentration Tyloxapol solutions. The number density of water in the core of the micelles in the different solutions is also reported, and we see a ten-fold increase in the amount of water in the core as the concentration increases from 5% w/w to 10% w/w.

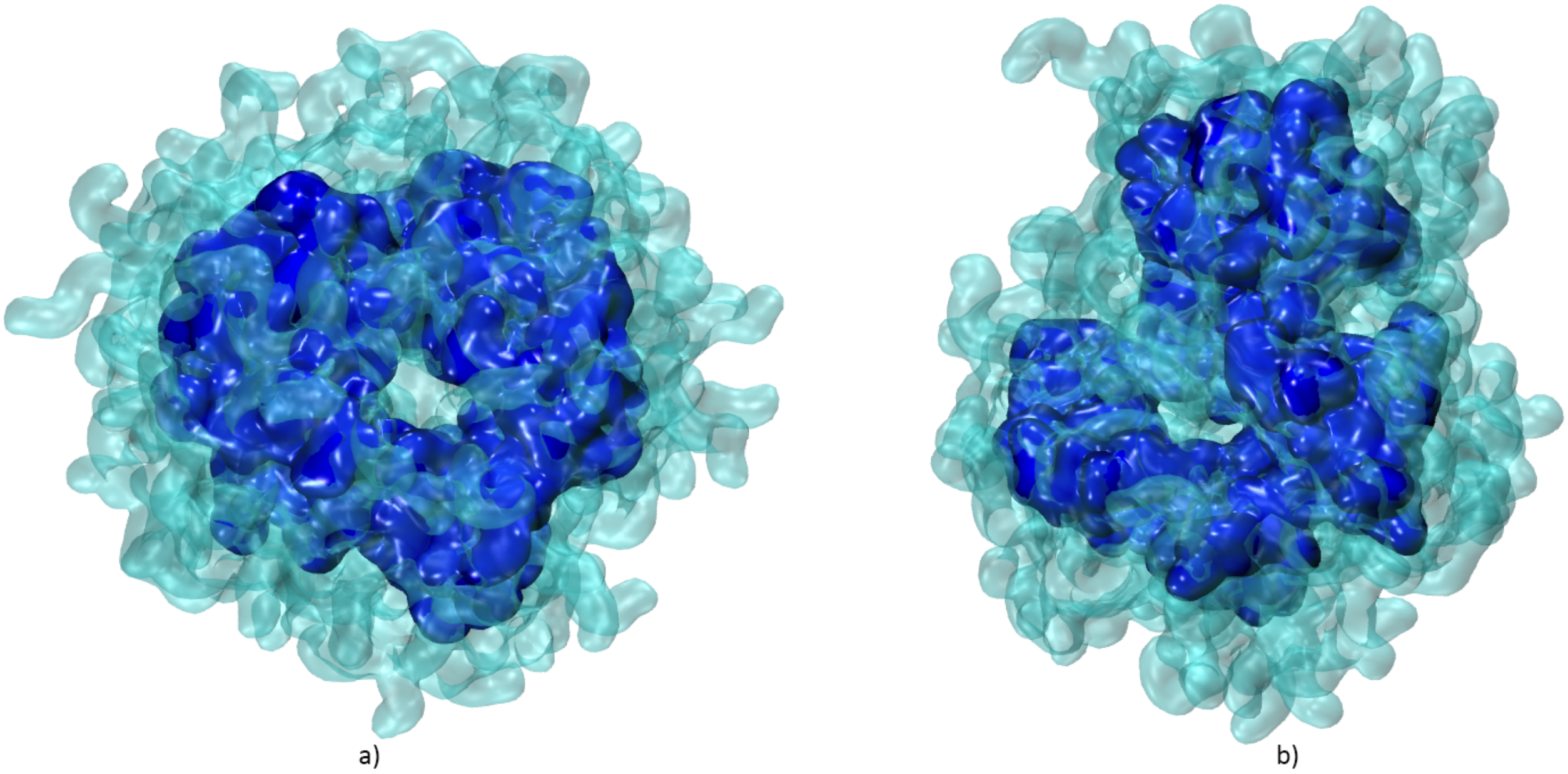

Figure S5: Pure a) TY39 and b) TY79 micelles. The corona and the hydrophobic core are colored in cyan and blue, respectively.

Table S1: **The core-shell interface surface area ($A_{\text{core-shell}}$), core volume (V_{core}), whole micelle volume (V_{micelle}) and the number of water molecules within the core of the micelles (n_w) as determined from the SANS data for the four different Tyloxapol solutions studied.**

Concentration (% w/w)	$A_{\text{core-shell}}$ (nm^2)	V_{core} (nm^3)	V_{micelle} (nm^3)	n_w (#/nm^3)
1	49.0	25.0	194.0	0.3
5	97.5	82.0	260.0	0.3
10	91.7	75.0	136.0	3.0
15	71.7	63.0	205.0	3.3

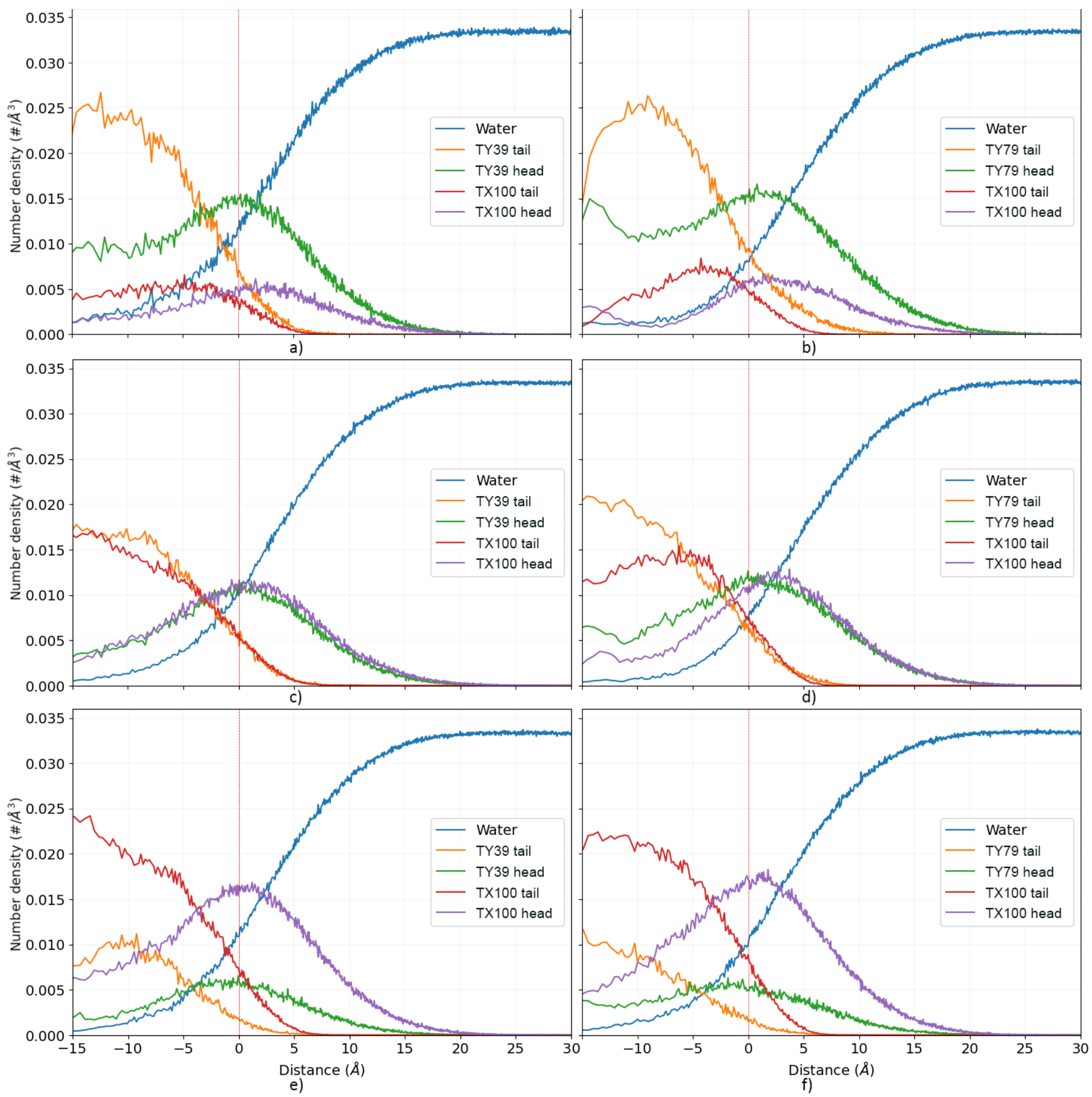

Figure S6: Densities of water, Tyloxapol and Triton x-100 in mixed systems. Left column shows Tyl3 systems.

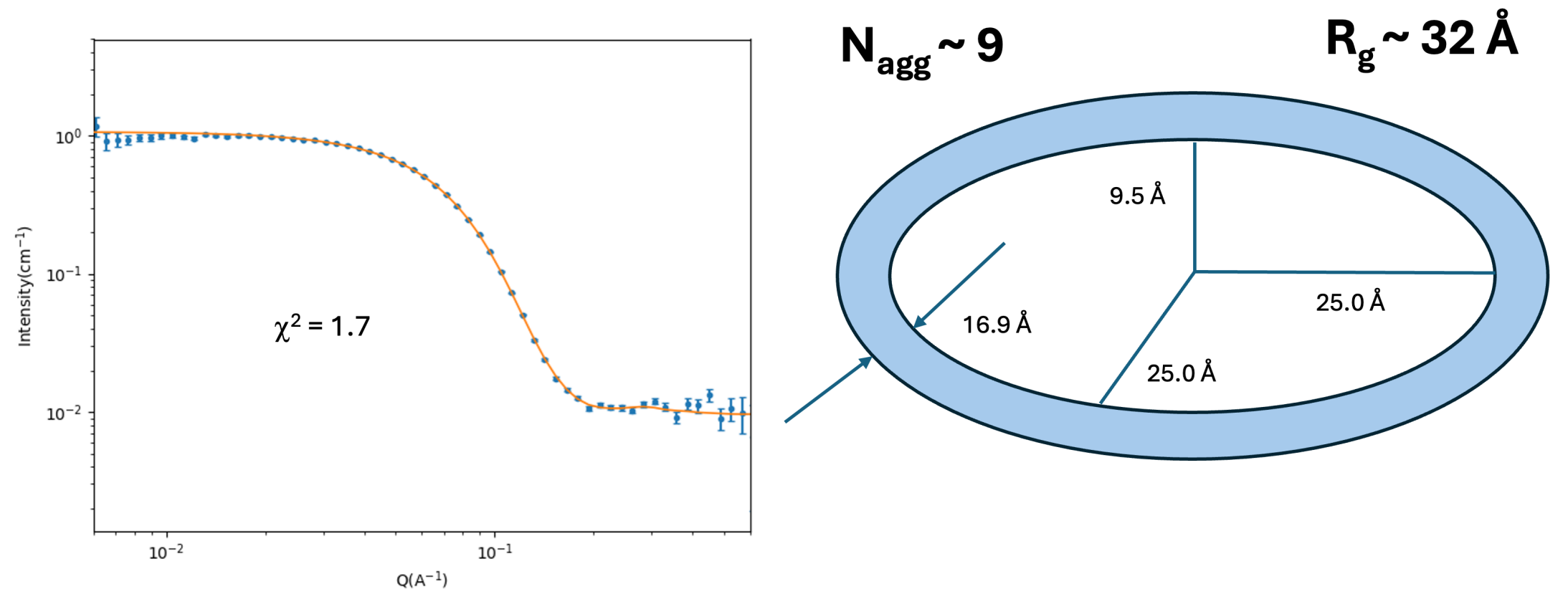

Figure S7: (left) The variation in the neutron scattering intensity as a function of the scattering vector (Q) for Tyloxapol micelles in a 1% w/w solution of Tyloxapol. The best fit to the SANS data (solid line) was obtained using a core–shell model for the form factor and a hard sphere structure factor to account for the interparticulate interactions. (right) Schematic showing the dimensions of the micelles as determined from the SANS scattering.

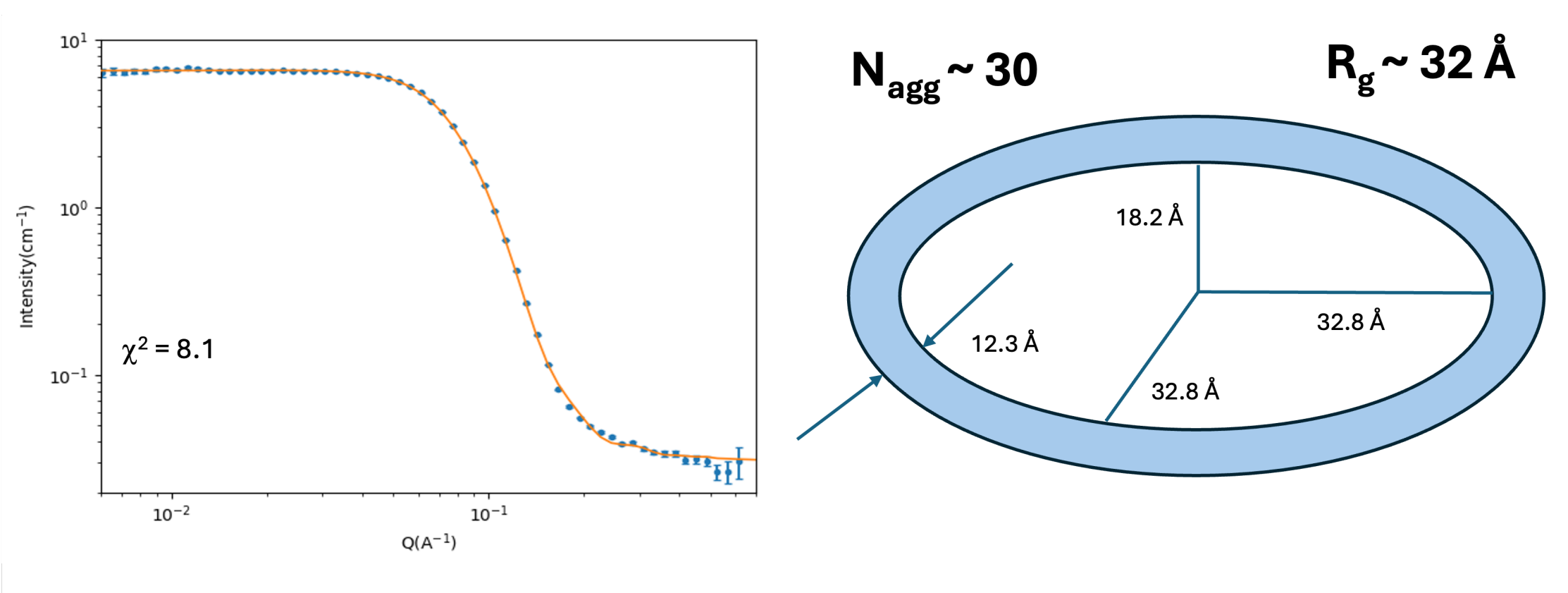

Figure S8: (left) The variation in the neutron scattering intensity as a function of the scattering vector (Q) for Tyloxapol micelles in a 5% w/w solution of Tyloxapol. The best fit to the SANS data (solid line) was obtained using a core–shell model for the form factor and a hard sphere structure factor to account for the interparticulate interactions. (right) Schematic showing the dimensions of the micelles as determined from the SANS scattering.

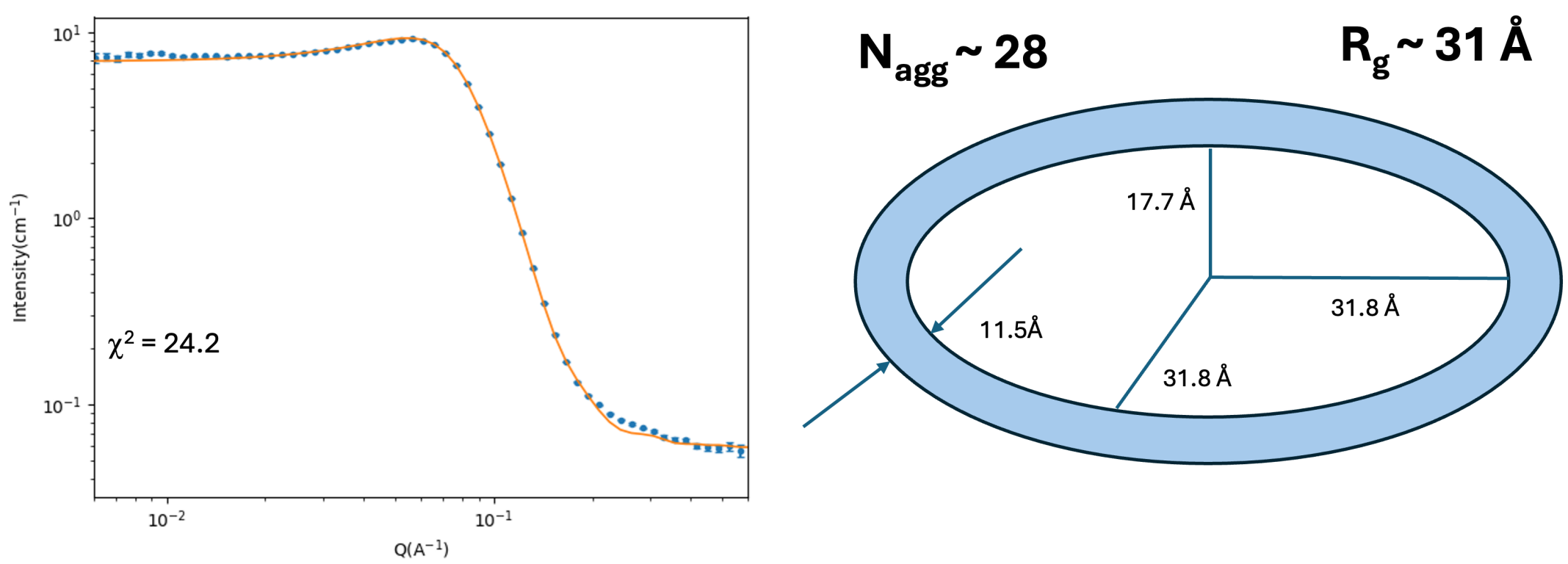

Figure S9: (left) The variation in the neutron scattering intensity as a function of the scattering vector (Q) for Tyloxapol micelles in a 10% w/w solution of Tyloxapol. The best fit to the SANS data (solid line) was obtained using a core–shell model for the form factor and a hard sphere structure factor to account for the interparticulate interactions. (right) Schematic showing the dimensions of the micelles as determined from the SANS scattering.
